# Live and inactivated *Piscirickettsia salmonis* activated nutritional immunity in Atlantic salmon (*Salmo salar*)

**DOI:** 10.3389/fimmu.2023.1187209

**Published:** 2023-04-28

**Authors:** Danixa Martínez, Ricardo Oyarzún-Salazar, Ana María Quilapi, José Coronado, Ricardo Enriquez, Carolina Vargas-Lagos, Cristian Oliver, Natacha Santibañez, Marcos Godoy, José Luis Muñoz, Luis Vargas-Chacoff, Alex Romero

**Affiliations:** ^1^ Laboratorio Institucional de Investigación, Facultad de Ciencias de la Naturaleza, Universidad San Sebastián, Puerto Montt, Chile; ^2^ Laboratorio de Inmunología y Estrés de Organismos Acuáticos, Facultad de Ciencias Veterinarias, Universidad Austral de Chile, Valdivia, Chile; ^3^ Escuela de Tecnología Médica, Facultad de la Salud, Universidad Santo Tomás, Osorno, Chile; ^4^ Centro de Investigaciones Biológicas Aplicadas (CIBA), Puerto Montt, Chile; ^5^ Centro de Investigación y Desarrollo i~mar, Universidad de los Lagos, Puerto Montt, Chile; ^6^ Instituto de Ciencias Marinas y Limnológicas, Facultad de Ciencias, Universidad Austral de Chile, Valdivia, Chile; ^7^ Centro Fondap de Investigación de Altas Latitudes (IDEAL), Universidad Austral de Chile, Valdivia, Chile; ^8^ Millennium Institute Biodiversity of Antarctic and Subantarctic Ecosystems, Biodiversity of Antarctic and Subantarctic Ecosystems (BASE), University Austral of Chile, Valdivia, Chile; ^9^ Centro Fondap Interdisciplinary Center for Aquaculture Research (INCAR), Universidad de Concepción, Concepción, Chile

**Keywords:** nutritional immunity, *Piscirickettsia salmonis*, *Salmo salar*, iron, zinc, manganese, Atlantic salmon

## Abstract

Nutritional immunity regulates the homeostasis of micronutrients such as iron, manganese, and zinc at the systemic and cellular levels, preventing the invading microorganisms from gaining access and thereby limiting their growth. Therefore, the objective of this study was to evaluate the activation of nutritional immunity in specimens of Atlantic salmon (*Salmo salar*) that are intraperitoneally stimulated with both live and inactivated *Piscirickettsia salmonis*. The study used liver tissue and blood/plasma samples on days 3, 7, and 14 post-injections (dpi) for the analysis. Genetic material (DNA) of *P. salmonis* was detected in the liver tissue of fish stimulated with both live and inactivated *P. salmonis* at 14 dpi. Additionally, the hematocrit percentage decreased at 3 and 7 dpi in fish stimulated with live *P. salmonis*, unchanged in fish challenged with inactivated *P. salmonis*. On the other hand, plasma iron content decreased during the experimental course in fish stimulated with both live and inactivated *P. salmonis*, although this decrease was statistically significant only at 3 dpi. Regarding the immune-nutritional markers such as *tfr1*, *dmt1*, and *ireg1* were modulated in the two experimental conditions, compared to *zip8*, *ft-h*, and *hamp*, which were down-regulated in fish stimulated with live and inactivated *P. salmonis* during the course experimental. Finally, the intracellular iron content in the liver increased at 7 and 14 dpi in fish stimulated with live and inactivated *P. salmonis*, while the zinc content decreased at 14 dpi under both experimental conditions. However, stimulation with live and inactivated *P. salmonis* did not alter the manganese content in the fish. The results suggest that nutritional immunity does not distinguish between live and inactivated *P. salmonis* and elicits a similar immune response. Probably, this immune mechanism would be self-activated with the detection of PAMPs, instead of a sequestration and/or competition of micronutrients by the living microorganism.

## Introduction

Aquaculture in Chile focuses on the industrial-scale production of salmonids fish, mainly Atlantic salmon (*Salmo salar*). However, the aquaculture companies suffer significant economic losses due to the high prevalence of infectious diseases, of which, Piscirickettsiosis caused by the bacterium *Piscirickettsia salmonis* is the most important disease (1, 2). Despite the fact that the identification of this bacterium dates back more than 30 years (3), the antimicrobial treatments so far were not effective in eliminating or in the least regulating the disease. It is estimated that this might be due to the ability of the bacterium to modulate the immune system of Atlantic salmon (*Salmo salar*) for the benefit of its permanence and replication (4). Similarly, the host-pathogen interaction modulates the regulation of micronutrients such as iron, as reported by Pulgar et al. (5), who demonstrated that families of Atlantic salmon (*Salmo salar*) that are resistant to infection with *P. salmonis* decrease the content of this micronutrient in the head kidney, compared to families with high susceptibility, suggesting that the regulation of iron-associated nutritional immunity could have a key role in fish survival.

Nutritional immunity involves the regulation of the availability of micronutrients such as iron, manganese, and zinc at systemic and cellular levels so that pathogenic microorganisms cannot access them (6). The activation of this mechanism has not only been evaluated in Atlantic salmon (*Salmo salar*) (5), and this mechanism has previously been described in Patagonian blennie (*Eleginops maclovinus*) challenged with *P. salmonis* (7, 8), as well as in Antarctic fish “*Notothenia rossii* – *Notothenia coriiceps*” stimulated with LPS (9) and, *in vitro* in Atlantic salmon head kidney cells (SHK-1) stimulated with several Pathogen-Associated Molecular Patterns (PAMPs) from *P. salmonis* (10). From a genomic point of view, orthologous genes involved in metabolism and iron uptake in *P. salmonis* have been identified as markers involved in the classical components of the tonB system, tonB-dependent siderophore synthesis/receptor, membrane exporters, ABC transporter, ferrous ion transport, tonB-dependent heme/siderophore receptor, and Fur family transcriptional regulator (5, 11, 12). In addition, it has been functionally demonstrated that *P. salmonis* can be cultured in artificial media supplemented with both ferric ammonium citrate and ferric nitrate inducing the synthesis of siderophores under experimental conditions (13).

The nutritional immunity also regulates other critical micronutrients such as manganese and zinc (6, 14). However, the uptake systems of these micronutrients by *P. salmonis* and how Atlantic salmon (*Salmo salar*) could regulate its homeostasis under infection conditions have not yet been investigated, although Pulgar et al. (5) reported no differences in the zinc content in the head kidney of Atlantic salmon (*Salmo salar*) between the families resistant and susceptible to *P. salmonis* were found after 14 dpi. Despite that micronutrients such as iron, manganese, and zinc can be captured from the water by fish (gills), the most important route of acquisition is from the diet (gastrointestinal tract) (15, 16). Thus, once assimilated they can be used in numerous biological functions within the cells (17, 18). Therefore, it is not surprising that their deficiencies or imbalances have repercussions on the immune response (19, 20) and much less that they are also considered micronutrients. that other bacteria require to induce their pathogenesis mechanisms (21–33).

Studies reveal that microorganisms have evolved sophisticated strategies to capture these micronutrients, and thus the proteins involved in their homeostasis in the host tissues must be correctly regulated. So far, iron-associated nutritional immunity has been evaluated in *S. salar* challenged with live *P. salmonis* (5) and in the SHK-1 cell line stimulated with outer membrane vesicles (OMVs), lipopolysaccharides (LPS) and total proteins (TP) of *P. salmonis* (10). Yet it is still unknown whether this antimicrobial defense mechanism discriminates between: live pathogens that require micronutrients and dead pathogens (inactivated bacteria) that do not. Therefore, the current research aimed to evaluate the activation of nutritional immunity through an *in vivo* approach using Atlantic salmon (*Salmo salar*) challenged with live and inactivated *P. salmonis* at different times post-injection (dpi).

## Material and methods

### Piscirickettsia salmonis

*P. salmonis* strain type LF-89 (ATCC VR-1361) was grown under standard conditions in Austral-SRS broth medium for 5 days at 18°C and 50 rpm of agitation (34). The identity was confirmed by Gram staining, polymerase chain reaction (PCR), and immunofluorescence antibody test (IFAT) (IFAT, SRS-Bios Chile), following the instruction manual. Subsequently, the inoculum used for the salmon treatment with inactivated *P. salmonis* was obtained by applying the previously described methodology for bacterial inactivation by thermal shock at 100°C for 30 min (35). The inactivation of the culture was confirmed by cultivating 100 μL of the inactivated inoculum in Austral-SRS broth solid medium without colony formation.

### Experimental challenge

Post-smolt specimens of Atlantic salmon (*Salmo salar*) (700 g) were obtained from local fish cultures (Valdivia, Chile) and transferred to Salmon Clinical Trials Facility, Institute of Animal Pathology, Faculty of Veterinary Medicine, Universidad Austral de Chile. The fish were kept in 1000 L ponds at 16°C, 12:12 photoperiod, 33 PSU salinity, 90-100% oxygen saturation, and fed once a day with Biomar pellets at 1% of their body weight (Proximate food analysis was 45-50% crude protein, 21-23% lipids, 9.5% carbohydrates, 12% ashes, 10% water, and 2.5% fiber). After the acclimation period, the fish (n=54) were randomly distributed into six 1000 L ponds for the implementation of the experimental treatments by intraperitoneal injection (i.p) and in duplicate: control (i.p fish with 100 μL of bacterial culture medium), live Ps (i.p fish with 100 μL of live *P. salmonis* at 1x10^4^ bact/μL) and inactivated Ps (i.p fish with 100 μL of inactivated *P. salmonis* at 1x10^4^ bact/μL). Three fish were randomly extracted from each pond (six for each treatment) at 3, 7, and 14 days post-injections (dpi) for blood/plasma and liver extraction. The bacterial dose applied was the same one used in a previous trial (5) and the experimental conditions in terms of temperature, salinity, oxygen, photoperiod and feeding were the same as those detailed in the acclimation period.

### Total DNA extraction

Total DNA extraction was from 60 mg of sample pool (liver) per experimental condition at 14 dpi, using a commercial Bacterial DNA isolation kit (Matchery-Nagel) and following the manufacturer’s instructions. Subsequently, the total DNA pellet was dissolved in diethylpyrocarbonate water, quantified by spectrophotometry (NanoDrop Technologies), and used for the detection of *P. salmonis* by qPCR with specific primers described in the literature (36). The last day (14 dpi) was selected to increase the sensitivity of the qPCR due to the bacterial load administered and the experimental times evaluated.

### Total RNA extraction

Total RNA was extracted from 50 mg of each tissue sample (liver) using the commercial kit E.Z.N.A ^®^ Total RNA Kit I (Omega) and Rnase-free Dnase I, following the manufacturer’s guidelines. Then the total RNA pellet was dissolved in diethylpyrocarbonate water, quantified by spectrophotometry (NanoDrop Technologies), and its quality was evaluated using 1% agarose gel. Subsequently, 1 μg of total RNA was used as a template for cDNA synthesis, using MMLV-RT reverse transcriptase (Promega) and oligo-dT primer (Invitrogen), according to the standard procedure (10).

### qPCR analysis

For the qPCR analysis, the QuantStudio™ equipment, Master Mix SYBRGreen (Life Technology, Thermo Scientific), and cDNA at 100 ng were used. Reactions were performed in triplicates in a total volume of 12 μL (6 μL SYBRGreen, 0.5 μL forward primers, 0.5 μL reverse primers, 3 μL PCR-grade water and 2 μL cDNA). Primers were designed for: transferrin receptor 1 (*tfr1*), divalent metal transporter 1 (*dmt1*), ferroportin 1 (*ireg1*), hepcidin (*hamp*), ferritin heavy-chain (*ft-h*), zinc transporter 8 (*zip8*), and small ribosomal subunit (*18s*) (10). The qPCR cycle conditions were 95°C for 10 min followed by 40 cycles of 95°C for 10 s and 60°C for 1 min. At the end of each reaction, the melting curve was evaluated to confirm the amplification and detection of a single PCR product, and the respective expression levels were analyzed using the comparative Ct method (2^-ΔΔCT^) (37). The data are presented as fold changes in gene expression normalized to an endogenous reference gene (*18s*) and relative to the uninfected fish (Control). The specific primers are listed in **Table 1**.

**Table 1:** Primer sequences.

Gene	Nucleotide sequences (5`→3`)	PCR product size (bp)	Accesion Number	References
*dmt1*	Fw: CGTCTTTTTCACGGGACAGC Rv: CGTACATGCATATAAATTGGTGGC	126	-	Martínez et al. (10)
*ft-h*	Fw: TCTGAACACAACGACCCACA Rv: GTCAAACAGGTACTCGGCCA	150	-	Valenzuela-Muñoz et al. (38)
*ireg1*	Fw: ACCACCGTGTAGCCCATTAAA Rv: TTGATAGCTAGCGGGCAGGA	105	XM_014173032.1	Martínez et al. (10)
*hamp*	Fw: GCCGATGCATTTCAGGTTCA Rv: AATGGCTTTAGTGCTGGCAGG	127	NM_001140849.1	Martínez et al. (10)
*tfr1*	Fw: GGGTCTAACTGGGAAGCAGC Rv: AACGGAATGAGACGGATGGG	100	XM_014188394.1	Martínez et al. (10)
*zip8*	Fw: ATGAACAGGACGGATCGACG Rv: AGCATTGGCTCTAACCCAGG	135	-	Martínez et al. (10)
*18s*	Fw: GTCCGGGAAACCAAAGTC Rv: TTGAGTCAAATTAAGCCGCA	116	AJ427629.1	Martínez et al. (10)
*P. salmonis*	Fw: AGGGAGACTGCCGGTGAT Rv: ACTACGAGGCGCTTTCTC	151	-	Karatas et al. (36)

### Plasma iron

Plasma iron levels were analyzed following the procedure indicated by Quilapi et al. (39), using the photometric technique of the Fer-color AA reaction kit (Wiener lab.) and the automatic biochemical analyzer (Erba XL-100). Briefly, 200 µL of plasma were mixed with 1 mL of reagent A (200 mM citric acid solution, 34 mM ascorbic acid, 100 mM thiourea and surfactant) and then, the absorbance was read by spectrophotometry at 600 nm. Subsequently, 200 µL of reagent B (ferene stabilized solution > 3 mM) were added to the previous mix and the absorbance was read after 5 minutes at 600 nm. The obtained absorbance is directly proportional to the iron concentration in the sample.

### Micronutrients in liver

The liver samples (1 g) were freeze-dried for 24 h in the Sumtrom model CT-FOL-12P freeze-dryer, belonging to the Center for Applied Biological Research (CIBA). Subsequently, they were sent to the Service Laboratory, Solespectro Ltda. (Santiago, Chile) for the quantification of micronutrients such as iron, manganese, and zinc using flame atomic absorption spectroscopy (FAAS). The concentration of micronutrients was expressed as µg metal/g DW.

### Statistical analysis

Two-way ANOVA was applied using injection type (Control, Live Ps, and Inactivated Ps) and time (3, 7, and 14 dpi) as variation factors. Data were transformed when necessary to meet parametric assumptions and *post hoc* Tuckey was applied to identify differences among groups. Each value is the mean ± S.E.M (n = 6). Different letters indicate statistical differences in the same treatment over time and symbols indicate statistical differences between the three conditions (Control, Live Ps, and Inactivated Ps) at the same time. Statistical differences were considered for a value of P < 0.05.

## Results

### Mortality and detection of *P. salmonis* DNA

**Figure 1:**
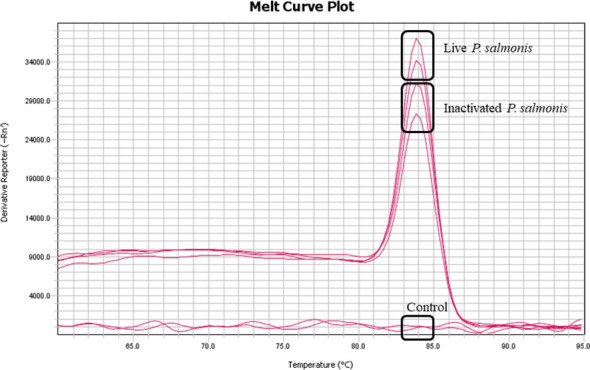
Detection of genetic material (DNA) of *P. salmonis* in the liver tissue at 14 dpi. Melting curve (Tm) of the *16s* gene from *P. salmonis* amplified by qPCR of pooled DNA from each experimental condition.

No mortality or behavioral changes were observed for any fish group during the experimental period (3-14 dpi). As estimated, the genetic material of *P. salmonis* (DNA) was detected at 14 dpi in the liver of fish stimulated with live and inactivated *P. salmonis*. No evidence of *P. salmonis* DNA was detected in the control group (**Figure 1**).

### Hematocrit

**Figure 2:**
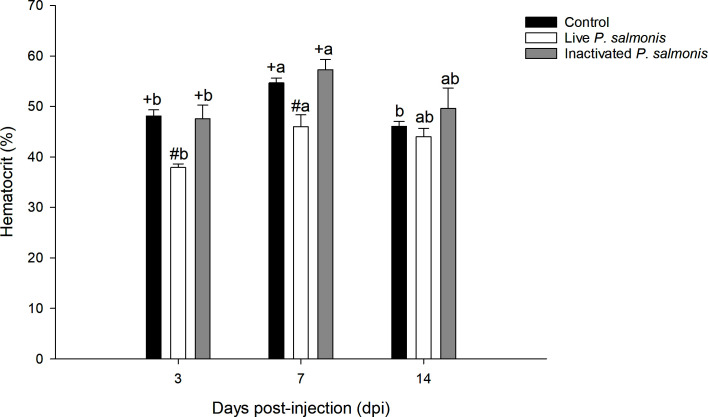
The hematocrit percentage in the blood of *S. salar*. The samples were taken at 3, 7, and 14 days after injection (dpi). Black bar: control condition, white bar (fish stimulated with live *P. salmonis*), and gray bar (fish stimulated with inactivated *P. salmonis*). Symbols (+, #) over the bars indicate statistical differences between the different treatments at the same time points. Different letters **(A, B)** indicate statistical differences in the same treatment at different times. Two-way ANOVA, p < 0.05; n=6.

The hematocrit percentage decreased in a statistically significant manner at 3 [37.89 ± 0.72] and 7 [46.0 ± 2.33] dpi in fish stimulated with live *P. salmonis*, compared to the control group [48.11 ± 1.21 (3 dpi)/54.68 ± 0.96 (7 dpi)]. On the other hand, no statistical differences between control fish group and the fish group stimulated with inactivated *P. salmonis* were observed. However, statistical differences between the different times for this treatment were determined (**Figure 2**).

### Plasma iron

**Figure 3:**
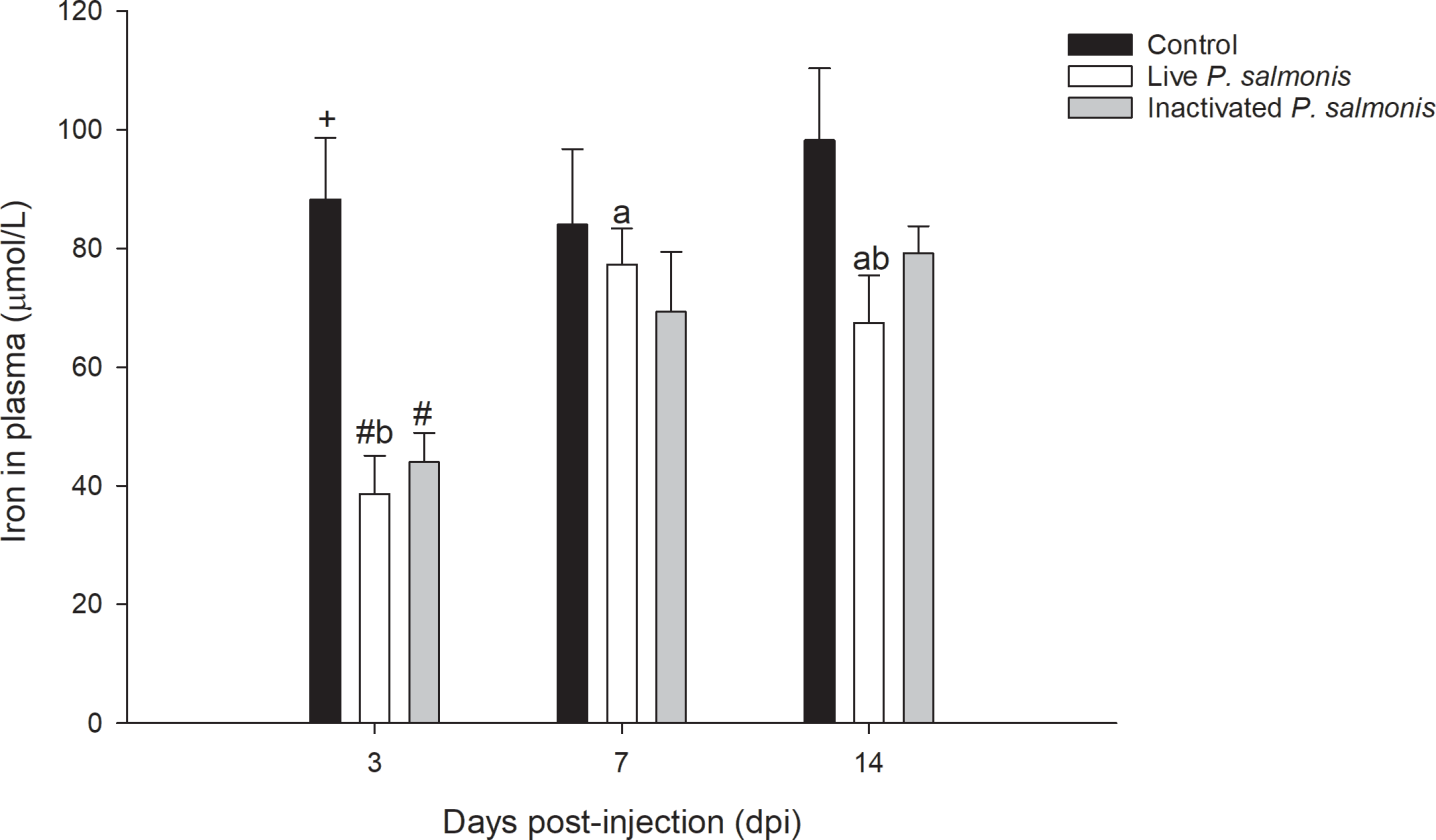
Plasma iron levels in *S. salar.* The samples were taken at 3, 7, and 14 days after injection (dpi). Black bar: control condition, white bar (fish stimulated with live *P. salmonis*), and gray bar (fish stimulated with inactivated *P. salmonis*). Symbol (#) over the bars indicates statistical differences between the different treatments at the same time points. Different letters a, b indicate statistical differences in the same treatment at different times. Two-way ANOVA, p < 0.05; n=6.

Plasma iron levels decreased statistically at 3 dpi in both the groups of fish stimulated with live *P. salmonis* [38.58 ± 6.48 µmol/L] and inactivated [43.97 ± 4.93 µmol/L], compared to the control group [88.28 ± 10.34 µmol/L]. In addition, a decrease in the iron levels at 7 and 14 dpi was also observed in both treatments; However, this decrease was not statistically significant (**Figure 3**).

### Gene expression of markers involved in nutritional immunology

**Figure 4:**
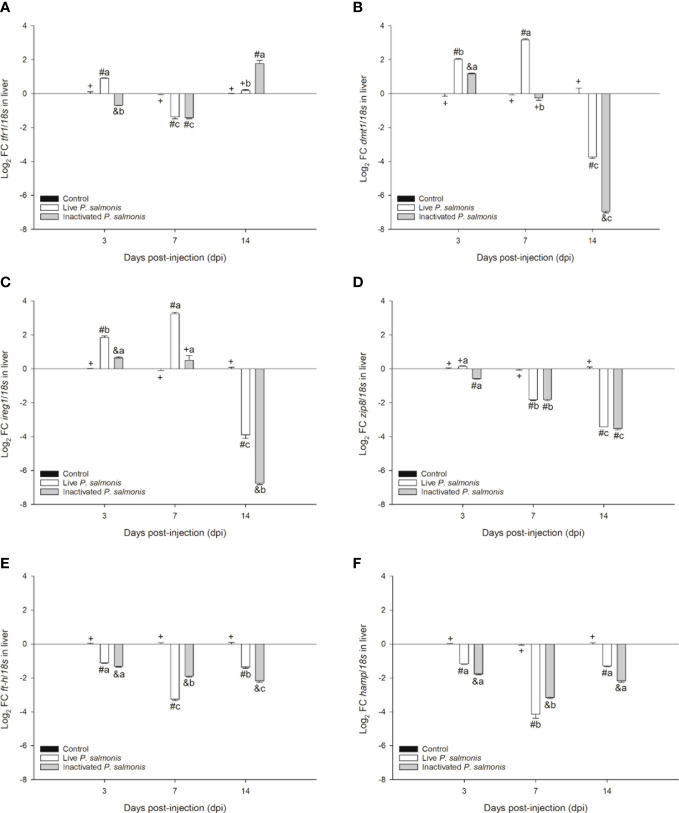
Gene expression of markers involved in nutritional immunology in *S. salar*. The samples were taken at 3, 7, and 14 days after injection (dpi). **(A)**: transferrin receptor 1. **(B)**: divalent metal transporter 1. **(C)**: ferroportin 1. **(D)**: zinc transporter 8. **(E)**: ferritin heavy chain. **(F)**: hepcidin. Symbols (+, #, &) over the bars indicate statistical differences between the different treatments at the same time points. Different letters a, b, c indicate statistical differences in the same treatment at different times. Two-way ANOVA, p < 0.05; n=6.

The *tfr1* marker involved in micronutrient uptake exhibited an increase in its transcription at 3 dpi [0.90 ± 0.03 Log_2_ FC] and decreased at 7 dpi [-1.36 ± 0.12 Log_2_ FC] in fish stimulated with live *P. salmonis*. On the contrary, fish stimulated with inactivated *P. salmonis* showed a decrease in the transcription of this marker at 3-7 dpi [-0.69 ± 0.02 (3 dpi)/-1.41 ± 0.06 (7 dpi) Log_2_ FC] with a statistically significant increase at 14 dpi [1.77 ± 0.18 Log_2_ FC] (**Figure 4A**).

Markers involved in the transport of micronutrients such as *dmt1* and *ireg1* displayed the same expression profile, i.e., increasing statistically at 3 dpi in fish stimulated with live [2.02 ± 0.04 (*dmt1*)/1.85 ± 0.09 (*ireg1*) Log_2_ FC] and inactivated *P. salmonis* [1.17 ± 0.04 (*dmt1*)/0.64 ± 0.07 (*ireg1*) Log_2_ FC]; however, at 7 dpi, the increase was statistically significant only in fish stimulated with live *P. salmonis* [3.17 ± 0.07 (*dmt1*)/3.23 ± 0.10 (*ireg1*) Log_2_ FC], compared to at 14 dpi, where the transcription of both markers decreased significantly in fish stimulated with live *P. salmonis* [-3.74 ± 0.07 (*dmt1*)/-3.89 ± 0.22 (*ireg1*) Log_2_ FC] and inactivated [-6.94 ± 0.09 (*dmt1*)/-6.74 ± 0.09 (*ireg1*) Log_2_ FC] (**Figures 4B, C**).

The *zip8* marker, which is also involved in the transport of micronutrients, significantly decreased its transcription in fish stimulated with live *P. salmonis* [-1.82 ± 0.03 (7 dpi)/-3.43 ± 0.05 (14 dpi) Log_2_ FC] and inactivated bacteria [-0.57 ± 0.02 (3 dpi)/-1.80 ± 0.07 (7 dpi)/- 3.52 ± 0.07 (14 dpi) Log_2_ FC]. At 3 dpi, fish stimulated with live *P. salmonis* did not show statistical differences compared to the control group (**Figure 4D**).

Finally, the markers involved in the storage (*ft-h*) and regulation (*hamp*) of micronutrients showed a statistically significant decrease in their transcription levels at 3, 7, and 14 dpi in fish stimulated with live *P. salmonis* [-1.11 ± 0.03 (3 dpi, *ft-h*)/-3.26 ± 0.08 (7 dpi, *ft-h*)/-1.36 ± 0.07 (14 dpi, *ft-h*)/-0.17 ± 0.02 (3 dpi, *hamp*)/-4.14 ± 0.23 (7 dpi, *hamp*)/-1.30 ± 0.04 (14 dpi, *hamp*) Log_2_ FC] and inactivated [-1.33 ± 0.03 (3 dpi, *ft-h*)/-1.90 ± 0.04 (7 dpi, *ft-h*)/-2.17 ± 0.09 (14 dpi, *ft-h*)/-1.77 ± 0.05 (3 dpi, *hamp*)/-3.16 ± 0.07 (7 dpi, *hamp*)/-2.17 ± 0.08 (14 dpi, *hamp*) Log_2_ FC] (**Figures 4E, F**).

### Micronutrients in liver

**Figure 5:**
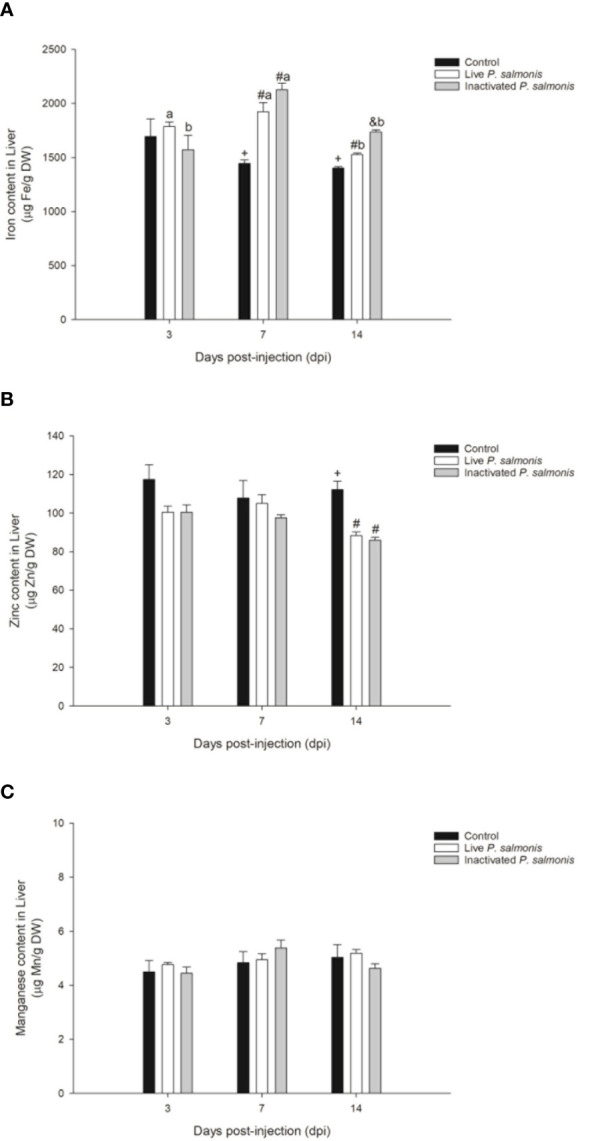
Micronutrient content in the liver of *S. salar*. The samples were taken at 3, 7 and 14 days after injection (dpi). Black bar: control condition, white bar (fish stimulated with live *P. salmonis*) and gray bar (fish stimulated with inactivated *P. salmonis*). **(A)**: iron. **(B)**: zinc. **(C)**: manganese. Symbols (+, #, &) over the bars indicate statistical differences between the different treatments at the same time points. Different letters a, b indicate statistical differences in the same treatment at different times. Two-way ANOVA, p < 0.05; n=6.

The concentration of iron in the liver tissue did not reflect any statistical differences with respect to the control group at 3 dpi; however, at 7 and 14 dpi, the tissue concentration of this micronutrient increased significantly in fish stimulated with live *P. salmonis* [1923.40 ± 85.07 (7 dpi)/1526.83 ± 13.92 (14 dpi) µg Fe/g DW] and inactivated [2127.00 ± 59.87 (7 dpi)/1736.00 ± 20.87 (14 dpi) µg Fe/g DW], compared to the control group [1444.00 ± 32.31 (7 dpi)/1402.83 ± 12.42 (14 dpi) µg Fe/g DW] (**Figure 5A**).

On the other hand, the zinc concentration did not show statistically significant changes at 3 and 7 dpi but decreased significantly at 14 dpi in fish stimulated with live *P. salmonis* [88.33 ± 1.89 µg Zn/g DW] and inactivated [86.00 ± 1.48 µg Zn/g DW], compared to the control group [112.16 ± 4.31 µg Zn/g DW] (**Figure 5B**).

Finally, the manganese content did not show statistically significant changes among the different treatments evaluated during the experimental course (**Figure 5C**).

## Discussion

This study evaluated the activation of nutritional immunity in Atlantic salmon (*Salmo salar*) specimens challenged intraperitoneally with *P. salmonis*, which causes high mortality rates in the Chilean salmon industry. We detected genetic material (DNA) of this bacterium in the liver of fish after 14 days of stimulation with both live and inactivated *P. salmonis*, indicating that the live bacterium could be established in this tissue, and that fish injected with the inactivated bacterium still retain remnants of genetic material that must be eliminated from their system. This may correlate with the bacterial dose administered (medium dose) into the tissue and the experimental time evaluated (3-14 dpi) since in fish the innate (early and non-specific) and adaptive (late and specific) immune responses have activation time ranges and both are necessary to combat pathogens and/or PAMPs at the cellular and systemic levels (40). In fact, Isla et al. (41) reported that IgM levels increase from day 28 in *S. salar* that are challenged with *P. salmonis*. Interestingly, an increase in IgM Anti-*P. salmonis* was observed from day 14 in *E. maclovinus* exposed to two strains of *P. salmonis* (42), suggesting that the immune response could be dependent on the species, stage of fish development, as well as doses and time of exposure to the pathogen.

Regarding the hematocrit, in the fish stimulated with live *P. salmonis*, it decreased in a statistically significant manner its percentage at 3 and 7 dpi while no changes were detected at 14 dpi. On the other hand, the fish stimulated with inactivated *P. salmonis* did not show changes in this parameter over the experimental course. The latter was to be expected since only a living pathogen provides its functional cellular machinery for the synthesis of hemolysins to be induced in *P. salmonis*, and consequently, the lysis of erythrocytes in Atlantic salmon *(Salmo salar)* (5), which could corroborate the anemia generated by Piscirickettsiosis (2). These results are not in agreement with what was previously published by Isla et al. (41), who reported a decrease in hematocrit from day 7 to 28 post-inoculation with live *P. salmonis*. However, this may be correlated to the bacterial dose they administered (low dose) and the size of the used smolt specimens (60-70 g), compared with the post-smolt specimens (700 g) used in this study, according to the ranges determined by Rozas-Serri et al. (43).

The lysis of erythrocytes is a mechanism of pathogenicity of *P. salmonis* that culminates in the release of iron that is contained in the heme group from these cell types (5). Interestingly, genomic (sequencing/annotation) and functional studies indicate that *P. salmonis* possesses the necessary machinery to sequester this micronutrient from different sources (5, 11–13, 44). Similarly, fish have developed mechanisms to avoid the sequestration of micronutrients, decreasing the levels of micronutrients at a systemic level (6). Our research demonstrated a decrease in the levels of plasma iron in fish stimulated with live and inactivated *P. salmonis* (3 dpi); however, this decrease was not statistically significant at 7 and 14 dpi. These findings agree with previous reports on smolt specimens of Atlantic salmon (*Salmo salar*) inoculated with a low dose of live *P. salmonis* (41) and in Patagonian blennie (*E. maclovinus*) challenged with *Francisella noatunensis* subsp. *Noatunensis* (39), which is a facultative intracellular bacterial pathogen that have similarities in terms of pathogenesis with *P. salmonis* (45).

The findings suggest that nutritional immunity is not capable of differentiating between live pathogens and dead or inactivated ones. Thus, the activation of this response could be induced by the detection of specific PAMPs rather than by the establishment of functional bacterial machinery. This was previously suggested in an *in vitro* assay using the SHK-1 cells stimulated with LPS, OMVs, and TP extracted from *P. salmonis*, where markers such as *zip8*, *zip14*, *dmt1*, *ireg1*, *tfr1*, *ft-h*, *ft-m*, *il6*, *hamp*, *irp1*, and *irp2* were modulated between 15 and 120 min post-stimulation with these different PAMPs (10), although in Antarctic fish such as *Notothenia rossii* and *Notothenia coriiceps*, the stimulation with commercial LPS did not alter plasma iron levels on day 5 post-intraperitoneal stimulation (9).

Immuno-nutritional markers that participate in the homeostasis of iron, manganese, and zinc are fundamental so that these are not sequestered by microorganisms (6, 14). In our research, these markers showed different expression profiles based on the type of bacterial stimulation (live and inactivated *P. salmonis*) and the experimental time evaluated (3-14 dpi). Specifically, *tfr1* involved in micronutrient uptake, showed a modulation in its transcription in fish stimulated with live and inactivated *P. salmonis*, while the transcription of the *dmt1* and *ireg1* transporters presented the same expression profile, increasing at 3 dpi and decreasing at 14 dpi in the fish stimulated with live and inactivated *P. salmonis*. On the other hand, *zip8* involved in the transport of Zn^2+^, Fe^2+^, HseO_3_^-^ y Mn^2+^ (46), *ft-h* involved in storage (47), and *hamp* involved in the regulation of micronutrients (48) showed down-regulation in their transcription levels in the liver of fish stimulated with both live and inactivated *P. salmonis* throughout the experimental course. The results suggest that stimulation with live and inactivated *P. salmonis* induces the transcriptional modulation of immune-nutritional markers in the liver of *S. salar* and that this modulation could be related to the decrease in plasma iron levels. These transcriptional findings have been corroborated by previous studies, which describe the activation of these markers in the head kidney of *S. salar* families with high and low susceptibility to *P. salmonis* (5), as well as in the liver and brain of *E. maclovinus* challenged with *P. salmonis* (7, 8), *F. noatunensis* subsp. *Noatunensis* (39), and in Antarctic fish such as Black rockcod (*Notothenia coriiceps*) and Marbled rockcod (*Notothenia rossii*) stimulated with commercial LPS (9).

Finally, the micronutrient content in the liver of Atlantic salmon (*Salmo salar*) revealed that the intracellular iron increased at 7 and 14 dpi in fish stimulated with live and inactive *P. salmonis*, while zinc content decreased at 14 dpi in both experimental conditions. However, stimulation with live and inactivated *P. salmonis* did not modulate the manganese content during the experiment. These results indicate that the decrease in iron in plasma could be related to the increase of this micronutrient in the liver and, more importantly, this mechanism could be a pathogenesis strategy used by *P. salmonis*, focused on concentrating the availability of intracellular iron necessary for its replication, similar to performed by *Francisella* spp (49). This was corroborated by Pulgar et al. (5), who indicated that families of Atlantic salmon (*Salmo salar*) susceptible to *P. salmonis* infection did not reduce the iron content in the head kidney unlike by the families resistant to this bacterial pathogen. In addition, these researchers concluded that the levels of zinc in the head kidney did not present any statistical differences between the families susceptible and resistant to *P. salmonis*. However, we observed a decrease in the zinc content at 14 dpi in the liver of fish stimulated with live and inactivated *P. salmonis*, which suggest that there could also be competition for this micronutrient, similar to that observed in other bacterial pathogens such as *Escherichia coli* (50), *Staphylococcus aureus* (51), *Salmonella typhimurium* (52) y *Mycobacterium tuberculosis* (53).

In conclusion, this study is the first to reveal the activation of nutritional immunity associated with micronutrient such as iron, manganese, and zinc at the systemic and cellular levels in *S. salar* when challenged with live and inactivated *P. salmonis*. The study results suggest that nutritional immunity in *S. salar* would not distinguish between live *P. salmonis* that needs micronutrients for its replication and inactivated *P. salmonis* which does not. The immune-nutritional markers involved in this response were modulated in both treatments, live and inactivated bacteria, during the experimental course (3, 7 and 14 dpi). This indicates that the nutritional immunity, being part of innate immunity, could be activated through the detection of PAMPs rather than a real sequestration and/or competition for the micronutrient by the live bacteria.

## Data availability statement

The original contributions presented in the study are included in the article/supplementary materials. Further inquiries can be directed to the corresponding authors.

## Ethics statement

The animal study was reviewed and approved by Ethical Committee of Universidad Austral de Chile (383/2020) in agreement with the National Agency for Research and Development (ANID, Chile).

## Author contributions

DM, LV-C, and AR conception and design of research; DM, RO-S, JC, and CV-L performed the experiments; DM, AQ, JC, and NS analyzed the samples; DM and RO-S analyzed the data; DM, RO-S, and CO interpreted the results of experiments; DM prepared the figures; DM drafted the manuscript; DM, MG, JM, LV-C, and AR edited and revised the manuscript; DM, RO-S, CO, and LV-C approved the final version of the manuscript.
